# Phytotoxic Effects and a Phytotoxin from the Invasive Plant *Xanthium italicum* Moretti

**DOI:** 10.3390/molecules17044037

**Published:** 2012-04-02

**Authors:** Hua Shao, Xiaoli Huang, Xiaoyi Wei, Chi Zhang

**Affiliations:** 1 Key Laboratory of Biogeography and Bioresource in Arid Land, Xinjiang Institute of Ecology and Geography, Chinese Academy of Sciences, Urumqi 830011, China; Email: shaohuaconnie@yahoo.com (H.S.); huangxl0430@163.com (X.H.); 2 South China Botanical Garden, Chinese Academy of Sciences, Guangzhou 510650, China; Email: xiaoywei@scbg.ac.cn; 3 State Key Laboratory of Desert and Oasis Ecology, Xinjiang Institute of Ecology and Geography, Chinese Academy of Sciences, Urumqi 830011, China

**Keywords:** phytotoxin, allelopathic, xanthinosin, invasive plant, *Xanthium italicum *Moretti

## Abstract

The allelopathic effects of different parts of the plant *Xanthium italicum* Moretti were evaluated by conducting bioassays against two dicot plants, amaranth (*Amaranthus mangostanus* L.) and lettuce (*Lectuca sativa* L.), and two monocot plants, wheat (*Triticum aestivum *Linn) and ryegrass (*Lolium multiforum*). Leaf and fruit extract possessed the strongest biological activity, killing all seeds of four test species at 0.05 g/mL concentration. Fruits were chosen for further investigation because of their high biomass. This led to the isolation and identification of a phytotoxin—Xanthinosin—a known sesquiterpene lactone. Xanthinosin significantly affected seedling growth of all test species at 160 µM concentration. Cultivating seeds in 800 μM xanthinosin solution resulted in a great decrease in seedling growth of all test species, especially for the two dicot plants, amaranth and lettuce, whose root length was inhibited by 78% and 89%, respectively. By comparison, the numbers were 69% lower for wheat, and 66% for ryegrass, two monocot plants. When treated with 4 mM xanthinosin solution, seed germination of all test plants was almost completely inhibited. The possibility of utilizing xanthinosin as an eco-friendly herbicide was discussed.

## 1. Introduction

**Figure 1:**
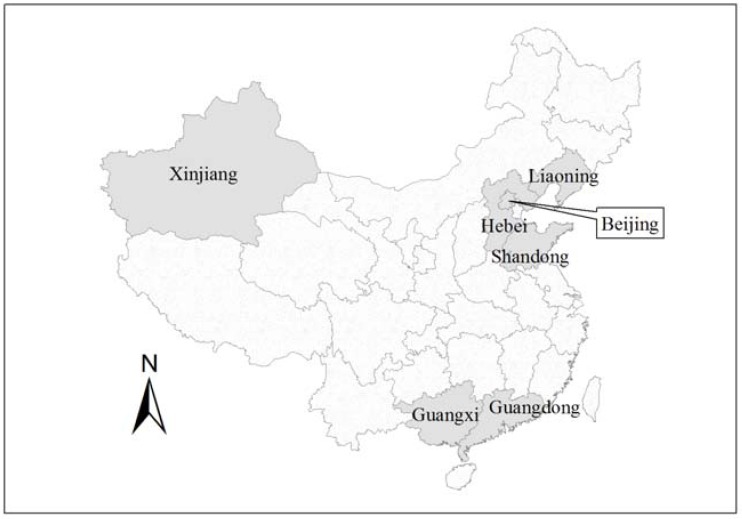
Provinces reporting the occurance of *X. italilcum *in China.

In China* Xanthium italicum* Moretti (family Asteraceae, Compositae) is a noxious annual invasive weed. Native to America, it was first spotted in Beijing, the capital of China in 1991 [1], and has spread quickly to six provinces over the past two decades [2,3]. In 2007, it was listed on the “People’s Republic of China imported plant quarantine pests list” [4]. Interestingly, all six provinces are located either on the coast or on the border (Figure 1), which is consistent with the hypothesis that *X. italicum* was transported to China via international trade [2]. Another interesting fact is that *X. italicum* is mainly distributed in three regions which are not only geographically far away from each other, but also characterized by distinctive climates: the northeastern region (which includes Liaoning, Hebei, Beijing and Shandong) has a temperate continental monsoon climate, the southeastern (Guangdong and Guangxi) is subtropical, and the northwestern region (Xinjiang) has a typical temperate continental arid/semiarid climate [5]. This phenomenon implies that *X. italicum* has the ability to easily adapt to different environmental conditions; it is also consistent with another report on the distribution of *X. italicum* in Japan, where this exotic plant was found to be dominant mainly in seaside habitats because it can tolerate salinity stress [6]. Although *X. italicum* is currently distributed in only a few places in China, ecological modeling has predicted that it has the potential to spread further to most regions in China, except for several areas with extreme environmental conditions such as Tibet [3]. 

Biological invasions cause great damage worldwide, especially to fragile ecosystems [7,8]. In the case of *X. italicum* invasion, we selected our study site in Xinjiang province, a typical arid region in far northwestern China on the border with Mongolia and Kazakhstan, where the World’s second largest desert—Taklamakan Desert—is located [5]. Arid ecosystems are relatively simple in structure and function compared with other terrestrial ecosystems, which makes them more susceptible to invasions [8,9]. The recent invasion of *X. italicum* has raised concerns regarding the possible ecological consequences it could bring to this arid land [3]; however, the mechanism underlying the successful invasion of this plant remains unclear. There have been many hypotheses attempting to elucidate the mechanism of exotic plant invasions [10]; among them, the “novel weapons hypothesis” has been controversial ever since it was proposed [11,12]. This hypothesis posits that some exotic invasive species owe their success to the production of novel allelochemicals that native species have never encountered [13,14,15]. Our previous study revealed that seedling growth of radish and wheat were significantly affected when grown in rhizosphere soil of *X. italicum*, indicating the possible involvement of allelochemicals which might act as a “novel weapon” in the invaded region and thus affect growth of native species (data not shown). Considering the fact that allelopathy is a notoriously difficult mechanism to demonstrate [11], we decide to start with the isolation and identification of phytotoxins in *X. italicum* plant which might work as potential allelochemicals after being released into the environment via leaching, root exudation or litter decomposition. Previous studies on other invasive plants have confirmed the presence of allelochemicals [16,17,18,19]; if we could verify the presence of potential allelochemicals at levels necessary to influence plant growth in soil beneath *X. italicum* plant, we believe we are at least getting closer to proving the contribution of allelopathy to the successful invasion of *X. italicum*. Moreover, if the putative allelochemicals don’t exist in *X. strumarium*, a native *Xanthium* plant, we could conduct further investigations which might consequently provide evidence to support the “novel weapons hypothesis”. 

## 2. Results and Discussion

### 2.1. Phytotoxicity Assays of Different Plant Parts of *X. italicum*

The strength of phytotoxicity of ethanol extracts on test plants varied greatly with the plant parts (Table 1). Leaf and fruit extracts exhibited the strongest biological activity on all test species compared with other extracts, completely inhibiting seed germination of all test species at 0.05 g/mL. Stem and root extract exhibited moderate to weak phytotoxic activity on four test plants, while litter extract showed the least effect. Given the fact that fruit has a much higher biomass than leaf, it was chosen for further investigation.

### 2.2. Phytotoxic Effects of Organic Extracts of *X. italicum* Fruit

Among all the organic extracts of *X. italicum* fruit, the chloroform extract consistently exhibited the most potent effect on four test species. Therefore, it was selected for further chromatographic separation. Amaranth seemed to be the most sensitive test species; petroleum ether and *n*-butanol extract significantly affected its seedling growth, meanwhile, their effects on the other three test species was much weaker (Table 2).

**Table 1:** Phytotoxic effects of different plant parts of *X. italicum* at 0.05g/mL.

	Amaranth		Lettuce		Wheat		Ryegrass	
	Root length	Shoot length	Root length	Shoot length	Root length	Shoot length	Root length	Shoot length
control	2.21 ± 0.26 ^a^	1.28 ± 0.08 ^a^	4.53 ± 0.36 ^a^	2.03 ± 0.06 ^a^	12.13 ± 0.56 ^a^	4.76 ± 0.05 ^a^	3.55 ± 0.10 ^a^	2.66 ± 0.06 ^a^
stem	1.00 ± 0.13 ^c^	1.29 ± 0.08 ^a^	1.83 ± 0.09 ^d^	2.25 ± 0.05 ^a^	8.63 ± 0.48 ^bc^	4.18 ± 0.03 ^b^	1.94 ± 0.22 ^c^	1.78 ± 0.20 ^bc^
leaf	0 ^d^	0 ^c^	0 ^e^	0 ^b^	0 ^d^	0 ^c^	0 ^d^	0 ^d^
fruit	0 ^d^	0 ^c^	0 ^e^	0 ^b^	0 ^d^	0 ^c^	0 ^d^	0 ^d^
root	1.00 ± 0.10 ^c^	0.89 ± 0.06 ^b^	2.41 ± 0.15 ^c^	1.98 ± 0.10 ^a^	7.74 ± 0.32 ^c^	4.43 ± 0.10 ^ab^	2.48 ± 0.21 ^b^	1.40 ± 0.15 ^c^
litter	1.63 ± 0.06 ^b^	1.34 ± 0.05 ^a^	3.78 ± 0.15 ^b^	2.08 ± 0.08 ^a^	9.92 ± 0.27 ^b^	4.22 ± 0.22 ^b^	2.60 ± 0.19 ^b^	1.98 ± 0.15 ^b^

Means within a column followed by the same letter are not different at *p *= 0.05 level according to Fisher’s LSD test. Each value is the mean of three replicates ± SE.

**Table 2:** Phytotoxic effects of organic extracts of *X. italicum* fruit at 1 mg/mL.

	Amaranth		Lettuce		Wheat		Ryegrass	
	Root length	Shoot length	Root length	Shoot length	Root length	Shoot length	Root length	Shoot length
control	2.21 ± 0.26 ^a^	1.28 ± 0.08 ^b^	4.53 ± 0.36 ^a^	2.03 ± 0.06 ^b^	12.13 ± 0.56 ^a^	4.76 ± 0.05 ^b^	3.55 ± 0.10 ^a^	2.66 ± 0.06 ^a^
Petroleum ether	1.15 ± 0.15 ^c^	0.71 ± 0.09 ^c^	4.85 ± 0.14 ^a^	2.23 ± 0.04 ^b^	8.14 ± 0.59 ^b^	4.37 ± 0.11 ^b^	3.14 ± 0.24 ^ab^	2.45 ± 0.17 ^a^
Chloroform	0.10 ± 0.00 ^e^	0.10 ± 0.10 ^d^	0.71 ± 0.08 ^c^	0.23 ± 0.05 ^d^	0.92 ± 0.13 ^c^	1.48 ± 0.12 ^d^	0.03 ± 0.03 ^d^	0.77 ± 0.15 ^bc^
Ethyl acetate	1.68 ± 0.18 ^b^	1.15 ± 0.14 ^b^	4.68 ± 0.18 ^a^	2.13 ± 0.04 ^b^	10.13 ± 0.29 ^a^	4.26 ± 0.15 ^b^	3.24 ± 0.10 ^a^	2.35 ± 0.09 ^a^
*n*-butanol	0.58 ± 0.11 ^d^	0.69 ± 0.09 ^c^	2.90 ± 0.08 ^b^	1.60 ± 0.04 ^c^	7.73 ± 0.15 ^b^	3.45 ± 0.07 ^c^	1.04 ± 0.06 ^c^	1.04 ± 0.10 ^b^
Water phase	1.58 ± 0.26 ^b^	2.39 ± 0.26 ^a^	3.25 ± 0.13 ^b^	3.35 ± 0.08 ^a^	11.34 ± 0.90 ^a^	5.52 ± 0.18 ^a^	2.88 ± 0.35 ^b^	2.35 ± 0.17 ^a^

Means within a column followed by the same letter are not different at *p *= 0.05 level according to Fisher’s LSD test. Each value is the mean of three replicates ± SE.

### 2.3. Identification of an Allelochemical from *X. italicum* Fruit

**Figure 2:**
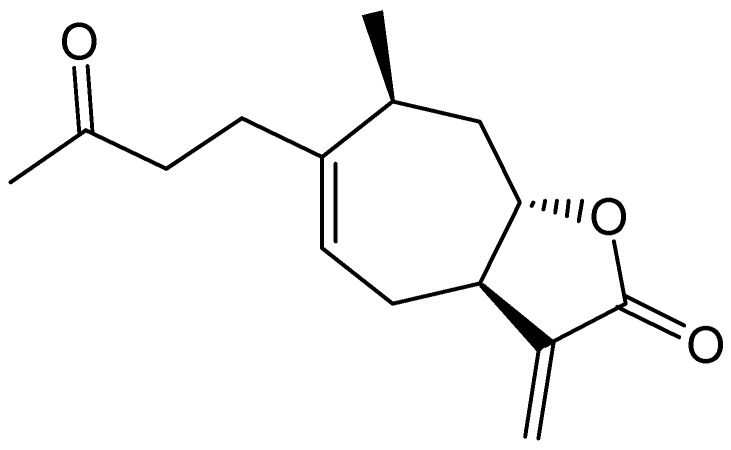
Chemical structure of xanthinosin.

The structure of the purified active compound was identified as xanthinosin by comparing its spectral data with published literature [20,21,22] (Figure 2). We obtained 820 mg of xanthinosin from 8 kg of dried *X. italicum* fruits (0.01% yield). Xanthinosin has been previously isolated from *X. italicum*, *X. macrocarpum *and *X. strumarium*, and it was confirmed to possess antileishmanial and antitumor activity [22,23,24]; however, no phytotoxic activity has ever been reported for it. Due to the fact that xanthinosin also exists in *X. strumarium*, a native *Xanthium* plant, our current study thus cannot provide any evidence to support the “novel weapons hypothesis”, although it is possible that *X. italicum* does produce unknown novel allelochemicals [25].

The genus *Xanthium* is the richest source of xanthanolides [26]. Besides xanthinosin, dozens of other xanthanolide lactones have been isolated from *Xanthium* plants, and many of them have been proven to possess a variety of biological activities including antimicrobial, anti-ulcerogenic, anti-inflammatory, antitumor and plant growth regulatory activity [23,27,28,29,30]. So far, the majority of reported chemical constituents of *X. italicum* are sesquiterpene lactones. *X. italicum* contains many other xanthanolide sesquiterpenes besides xanthinosin. Kovacs *et al*. isolated four xanthanolides, *i.e.*, xanthatin, 4-epixanthanol, 4-epi-isoxanthanol, and 2-hydroxyxanthinosin, from leaves of *X. italicum* with antitumor activity [29]. Marco *et al*. identified five xanthanolides from aerial parts of *X. italicum* including xanthinosin and 4-epixanthanol [22]. Tsankova obtained six xanthanolides from leaves of *X. italicum*, mainly xanthinin and xanthatin [30]. It is noteworthy to mention that xanthinin was reported to possess plant growth regulatory effects [31]. Another xanthanolide, 8-*epi*-xanthatin, which doesn’t exist in *X. italicum*, also exhibited plant growth regulatory activity [32]. In case both xanthinosin and xanthinin (or other xanthanolides, too) can be released to the environment and persist in soil at effective concentration for a certain period of time to affect other plants’ growth, it is necessary to investigate whether these co-occurring sesquiterpenoids could result in synergistic effects [33]. Although xanthinosin (possibly xanthinin and 8-*epi*-xanthatin, too) has the potential to be exploited as an environmentally friendly herbicide, still, there is a long way to go before it can be successfully commercialized. There are some issues that limit the utilization of naturally occurring substances directly as herbicides including their low yields, weak inhibitory activity, high toxicity, instability, short persistence in soil, *etc*.; on the other hand, there are successful cases of using natural products as new lead compounds to synthesize commercial herbicides. Examples include mesotrione, a synthesized analogue of leptospermone which is produced by the roots of the “bottle brush” plant *Callistemon citrinus *[34], and cinmethylin, a derivative of 1,4-cineole which is a natural phytotoxin found in the essential oils of a number of plants [35].

### 2.4. Phytotoxic Effects of Purified Xanthinosin

**Figure 3:**
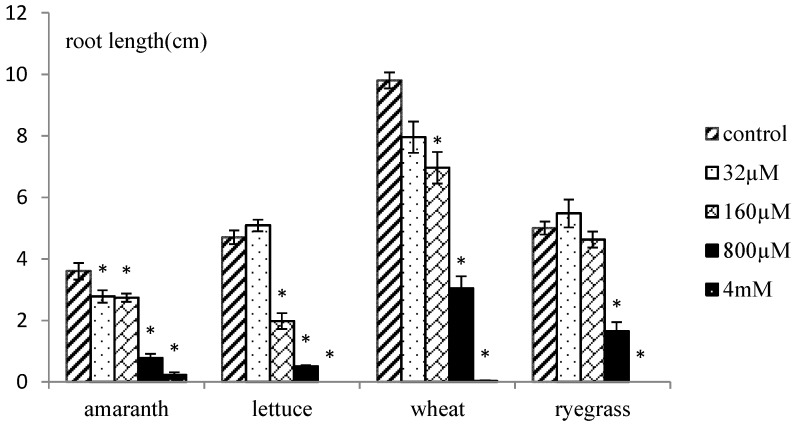
Phytotoxic effects of xanthinosin on root growth of amaranth, lettuce, ryegrass and wheat. Significant differences with the control treatment (*p* < 0.05, LSD) are indicated by *.

**Figure 4:**
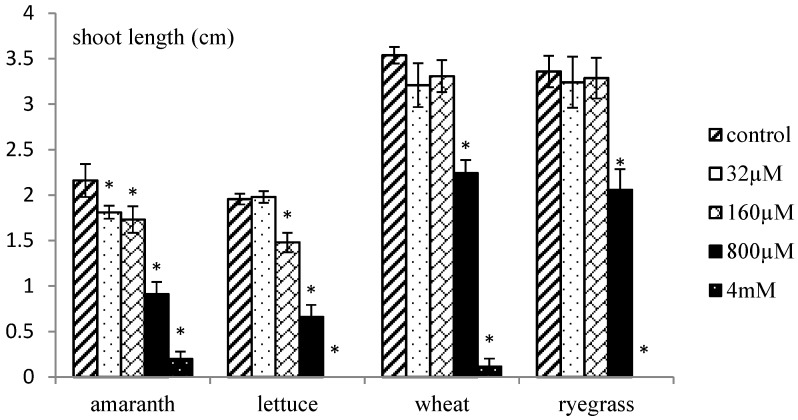
Phytotoxic effects of xanthinosin on shoot growth of amaranth, lettuce, ryegrass and wheat. Significant differences with the control treatment (*p* < 0.05, LSD) are indicated by *.

Xanthinosin didn’t affect seedling growth of any test plants when the concentration was low (32 µM). From 160 µM, significant inhibitory effects were observed on most test plants, especially lettuce, whose root elongation was inhibited by 58%. When treated with 800 µM xanthinosin solution, two dicot plants, *i.e.*, amaranth and lettuce, seemed to be more sensitive than two monocot species, wheat and ryegrass. Root growth of amaranth and lettuce was inhibited by 78% and 89%, respectively, while the numbers were 69% lower for wheat, and 66% for ryegrass (Figure 3). When the concentration reached 4 mM, seed germination of all test species was mostly completely inhibited. Shoot growth of all species responded to xanthinosin in a similar way, but to a lesser extent (Figure 4).

## 3. Experimental

### 3.1. General

IR spectra were measured on a Perkin-Elmer 783 spectrometer with KBr disks. UV spectra were taken on a Perkin Elmer Lambda 25 UV/VIS spectrometer. ^1^H- and ^13^C-NMR spectra were recorded on a Varian Unity Inova instrument (at 400/100 MHz). EIMS were measured on a Micromass Platform EI-200 GC/MS instrument at 70 eV by direct insertion probe.

### 3.2. Plant Material

Plants and fresh litter of *X. italicum* were collected randomly from a roadside monoculture in Changji city, Xinjiang province in September, 2011 (N43°54′40.2″, E87°17′7.6″). Plants were identified by Professor Wenfang Wang from Linyi University. The plant materials were air dried in our laboratory at room temperature for two weeks. 

### 3.3. Phytotoxic Effects of Ethanol Extracts of Different Plant Parts

Two dicot plants, amaranth and lettuce, and two monocots, wheat and ryegrass, were chosen as test plants for this assay. Seeds were surface sterilized with 0.5% HgCl_2_ before use. Air dried plants of *X. italicum* were separated into stems, leaves, fruits, roots and litter. Five g of each part were ground into powder and soaked in 80% ethanol (100 mL) for 24 h to afford 0.05 g/mL extract. Three mL of ethanol extract of each plant part were then added to Petri dishes (9 cm diameter) lined with Whatman No. 3 filter paper. After complete evaporation of ethanol, distilled water (3 mL) was added to each Petri dish followed by addition of 10 seeds. Petri dishes were sealed with Parafilm to prevent water loss and stored in the dark at 25 °C. Seedlings were measured after 4 days of cultivation for amaranth, lettuce and wheat, and 5 days for ryegrass. Three replicates were made for all phytotoxic bioassays.

### 3.4. Phytotoxic Effects of Organic Extracts of Fruits

Fruits of *X. italicum* were selected for further isolation because of their strong phytotoxic activity and high biomass. Two hundred g of dried fruits of *X. italicum* were first extracted in ethanol for a week and then subjected to solvent-solvent partition using the following organic solvents sequentially: petroleum ether, chloroform, ethyl acetate and *n*-butanol, to afford corresponding extracts. The extracts along with the remaining water phase were filtered and evaporated to dryness *in vacuo*, weighed, and redissolved in 95% ethanol (for organic extracts) or distilled water (for the water phase) to give 1 mg/mL solutions. They were then tested for their phytotoxic effects at 1 mg/mL concentration using the same method for plant part extracts. Again, amaranth, lettuce, wheat and ryegrass served as test plants.

### 3.5. Extraction and Isolation

Eight kg of dried fruits were ground into powder and exhaustively extracted with 80% ethanol at room temperature for three weeks (3 × 40 L). The ethanol extract was concentrated under reduced pressure to yield a dark brown residue which was subsequently subjected to solvent-solvent partition into petroleum ether, chloroform, ethyl acetate, and *n*-butanol subfractions. Results from the bioassays indicated that the chloroform extract possessed the strongest inhibitory effect. Thus 10 g chloroform extract were fractionated on silica gel eluted with a step gradient elution (CHCl_3_, CHCl_3_-MeOH at 98:2, 96:4, 9:1, 8:2, 7:3, 6:4, 1:1, MeOH). Twenty-four major fractions were collected based on TLC profiles; they were all subsequently assayed for phytotoxic activity on lettuce at 1 mg/mL. Among them, fraction 5 and 6 completely inhibited seed germination of lettuce, and they were chosen for further study. Fractions 9–10, 16–17 also exhibited moderate biological effects. However, considering the low yield of these fractions, we gave up on them. Fraction 5 was then further purified by Sephadex LH-20 to afford an oily liquid that was later identified as xanthinosin; fraction 6, which contains lots of impurities, was subjected to silica gel column chromatography, after which we lost track of the active ingredient and thus failed to identify it in this fraction.

### 3.6. Phytotoxic Effects of Purified Xanthinosin

Phytotoxic activity of purified xanthinosin obtained from the above purification procedure was assessed by conducting bioassays with similar procedures at 8, 40, 200, 1,000 µg/mL concentrations against the same four test plants.

### 3.7. Statistical Analyses

The significance of effects of extracts of *X. italicum* as well as xanthinosin on seedling growth of test species was first examined by ANOVA (*p* < 0.05) and then analyzed using Fisher’s LSD test at *p* < 0.05 level.

## 4. Conclusions

We isolated a phytotoxin—Xanthinosin—from fruits of *X. italicum*, which exhibited phytotoxic activity on four test plants. Further research is needed to explore the possibility of utilizing this compound as an environmentally friendly herbicide.
